# CD38 Multi-Functionality in Oral Squamous Cell Carcinoma: Prognostic Implications, Immune Balance, and Immune Checkpoint

**DOI:** 10.3389/fonc.2021.687430

**Published:** 2021-06-15

**Authors:** Zhuang Ding, Yijia He, Yong Fu, Nisha Zhu, Mengxiang Zhao, Yuxian Song, Xiaofeng Huang, Sheng Chen, Yan Yang, Caihong Zhang, Qingang Hu, Yanhong Ni, Liang Ding

**Affiliations:** ^1^ Central Laboratory of Stomatology, Nanjing Stomatological Hospital, Medical School of Nanjing University, Nanjing, China; ^2^ Department of Oral Pathology, Nanjing Stomatological Hospital, Medical School of Nanjing University, Nanjing, China; ^3^ Research Institute of Superconductor Electronics, School of Electronic Science and Engineering, Nanjing University, Nanjing, China

**Keywords:** CD38, oral squamous cell carcinoma, prognosis, lymphocyte subsets, immune checkpoint

## Abstract

**Background:**

CD38 belongs to the ribosyl cyclase family and is expressed on various hematological cells and involved in immunosuppression and tumor promotion. Although targeting CD38 antibodies has been approved for treatment of multiple myeloma, the function of CD38 in solid tumor, oral squamous cell carcinoma (OSCC) *etc.*, has not been investigated.

**Methods:**

This retrospective study included 92 OSCC samples and analyzed the spatial distribution of CD38 by immunohistochemistry (IHC). The values of diagnosis and prognosis of CD38 were evaluated. Additionally, 53 OSCC preoperative peripheral blood samples were used to be analyzed by flow cytometry. Tumor Immune Estimation Resource (TIMER) and cBioPortal databases were used to study CD38 level in various tumors and its correlation with tumor immune microenvironment in head and neck squamous cell carcinoma (HNSCC).

**Results:**

CD38 ubiquitously presented in tumor cells (TCs), fibroblast-like cells (FLCs), and tumor-infiltrating lymphocytes (TILs). Patients with highly expressed CD38 in TCs (CD38^TCs^) had higher TNM stage and risk of lymph node metastasis. Upregulation of CD38 in FLCs (CD38^FLCs^) was significantly associated with poor WPOI. Escalated CD38 in TILs (CD38^TILs^) led to higher Ki-67 level of tumor cells. Moreover, patients with enhanced CD38^TCs^ were susceptible to postoperative metastasis occurrence, and those with highly expressed CD38^TILs^ independently predicted shorter overall and disease-free survival. Strikingly, patients with highly expressed CD38^TILs^, but not CD38^TCs^ and CD38^FLCs^, had significantly lower CD3^+^CD4^+^ T cells and higher ratio of CD3^−^CD16^+^CD56^+^NK cells. The imbalance of immune system is attributed to dysregulated immune checkpoint molecules (VISTA, PD-1, LAG-3, CTLA-4, TIGIT, GITR) as well as particular immune cell subsets, which were positively correlated with CD38 expression in HNSCC.

**Conclusion:**

CD38 is a poor prognostic biomarker for OSCC patients and plays a vital role in governing immune microenvironment and circulating lymphocyte homeostasis. Co-expression between CD38 and immune checkpoint molecules provides new insight into immune checkpoint therapy.

## Introduction

Squamous cell carcinoma is the main malignancy of oral mucosa and lip, accounting for 90–95% of malignancies in these parts. This is the sixth largest malignancy in the world (1). Distant metastasis plays a critical role in the management and 5-year overall survival rate of treated patients with OSCC (2). Therefore, identification of valuable molecular markers for diagnosis and individualized treatment is critical for improving the prognosis of OSCC patients.

CD38 (cluster of differentiation 38) is a 45 KD single chain type II transmembrane glycoprotein, encoded by human chromosome 4p15 (3). CD38 was observed in inner membranes such as the endoplasmic reticulum, nuclear membrane, and mitochondria (4). Functionally, CD38 is initially considered a biomarker to identify activated T cell and thymocytes (5). Moreover, CD38, the main cellular NADase in mammalian tissues, has both hydrolysis and cyclization functions. The catalytic products of CD38 play an important role in extracellular metabolites, intracellular calcium ions, cell adhesion, signal transduction pathways, tumorigenesis and immunosuppression (6). The receptor/ligand activity of CD38 has been confirmed in various immune cell types, and its function in lymphocyte development, activation and differentiations are different (7), but the role of CD38 in solid tumor is far from known.

Of note, as a pro-tumoral molecule, CD38 has been paid more attention with the development of research (8). CD38 is linked to a more malignant clinical behavior, thus emerged as an unfavorable prognostic marker for CLL patients (9). Higher CD38^+^ clone CLL cells were more sensitive to BCR signal and have the characteristics of enhanced migration (10). CD38 expression in colorectal cancer was enhanced on monocytic myeloid-derived suppressor cells (M-MDSCs). These CD38^+^ M-MDSCs possessed a greater capacity to suppress the T cell proliferation by a mixed lymphocyte reaction assay, which contributed to colorectal cancer (CRC) pathogenesis. Interestingly, CD38^+^M-MDSCs were amplified in the peripheral blood of CRC patients who had a history of treatment, indicating that CD38^+^M-MDSCs could participate in treatment resistance (11). In addition, CD38 mRNA and protein were found to be over-expressed in human lung cancer cell lines and lung cancer specimens. The knockout of CD38 inhibited anchorage-independent cell growth, cell invasion, and significant reduction of tumor growth (12). Until now, the expression pattern and function of CD38 in OSCC has remained unexplored.

Therefore, in this study, we detected the expression pattern of CD38 protein in OSCC, including TCs, FLCs, and TILs. We further evaluated the correlation between CD38 and clinicopathological features and their prognostic value. Considering the important role of CD38 in immune cells, we also assessed the relationship between CD38 and peripheral blood lymphocyte subsets as well as tumor infiltrating immune cells. Additionally, cancer cells could use immune-checkpoint pathways as a major mechanism of immune resistance. Immune-checkpoint therapies have attracted wide attention because of its clinical potential to improve durable outcomes of cancer patients. However, CD38 overexpression was reported to contribute to resisting checkpoint inhibitors (13). Thus, we further studied the association between CD38 and immune checkpoint molecules.

## Materials and Methods

### Patients and Samples

All the schemes of this study were examined and approved by the Ethics Committee of Nanjing Stomatology Hospital. The study was carried out in accordance with the Declaration of Helsinki. From 2007 to 2016, 92 primary OSCC patients were enrolled. The inclusion and exclusion criteria of patients were the same as those of our previous studies (14). These patients were followed up for 3–60 months, and the median was 36 months. Paraffin-embedded OSCC tissue slices were obtained from the pathology department and used for IHC study. 53 blood samples from OSCC patients were obtained for flow cytometry assay before any related treatments.

### Immunohistochemistry and Quantification

The protocol of IHC of formalin-fixed paraffin-embedded sections and scoring details of IHC was performed as previously described (15). Anti-CD38 (25284-1-AP; Proteintech) and anti-Ki-67 (ab16667; Abcam) were used at a dilution ratio of 1:300 and 1:200 respectively. We used PBS to replace the primary antibody as negative control. The IHC staining results of CD38 and Ki-67 were evaluated by two senior pathologists who did not know the patients data, and the average values were calculated for further analysis. Due to insufficient tissues and individual differences in OSCC samples, certain regions or cell types, such as FLCs and TILs, could not be detected in the IHC staining. The expression levels of CD38 in TCs and FLCs, and Ki-67 in tumor cells were defined as “low” when it is lower than the average value and as “high” when it is equal to or greater than the average. When it is lower than the upper quartile, the expression level of CD38 in TILs was defined as “low”, and when it is equal to or greater than the upper quartile, it is defined as “high”.

### Flow Cytometry Assay

For the cell subtypes of peripheral blood mononuclear cells (PBMCs) analysis, details of this protocol were the same as the previous description (16).

### Gene Correlation Analysis in cBioportal

The cBioPortal for Cancer Genomics (http://cbioportal.org) is a website for exploration of multi-dimensional cancer genomics data, providing readily understandable gene expression event (17). We used cBioportal to analyze the correlation between CD38 and specific lymphocyte subset markers as well as specific immune checkpoint molecules in HNSCC. Co-expression was calculated based on the cBioPortal’s online instructions.

### TIMER Analysis

TIMER (https://cistrome.shinyapps.io/timer/) is a user-friendly website for cancer researchers to evaluate the comprehensive correlation analysis between tumor-infiltrating immune cell markers and selected genes. We used TIMER to assess the difference of CD38 between tumors and adjacent normal tissues. In addition, according to the online instructions of TIMER, we also assessed the correlation between the CD38 and markers of specific immune infiltrating cell subset at transcription level.

### Statistical Analysis

SPSS 22.0 and GraphPad Prism 8.0 software packages were used for data analysis and graphic processing. Pearson’s chi-square test, Fisher’s exact test and Chi-square test were used to compare clinicopathological features. The Mann–Whitney U test was used to compare the two groups. Survival analysis includes overall survival (OS) and disease-free survival (DFS), which were evaluated by Kaplan–Meier and log-rank test. Further multivariate analysis was carried out by Cox proportional hazards regression model to determine the independent risk factors, adjusted hazard ratio (HR) and 95% confidence interval (CI) of OSCC. Wilcoxon test was used to compare differential expression of CD38 between tumors and adjacent normal tissues. Co-expression between CD38 and immune cell markers and immune checkpoint molecules was investigated by Pearson correlation analysis. The partial Spearman’s correlation analysis was used to analyze the association between CD38 and markers of specific immune infiltrating cell subset at transcription level. All statistical tests were two-sided, and p <0.05 was considered to be significant.

## Results

### CD38 Was Ubiquitously Expressed in TCs, FLCs and TILs in OSCC

**Figure 1:**
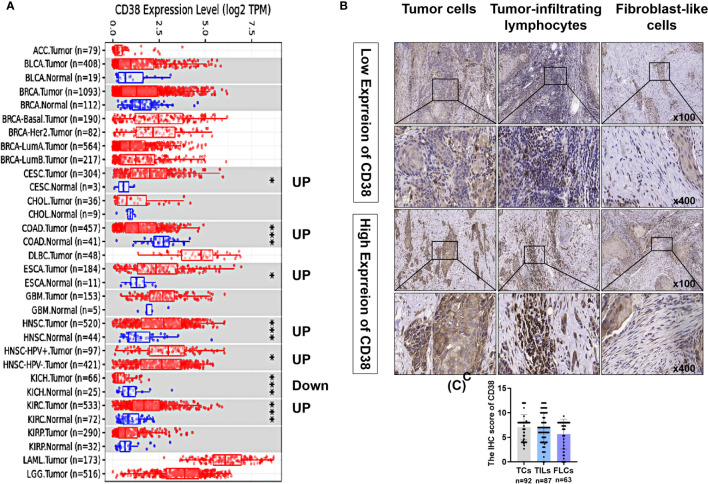
Expression levels of CD38 in OSCC and other cancers. **(A)** The CD38 expression in different tumor types with or without normal tissues with TIMER and the statistical significance was marked by the number of stars. **(B)** Typical IHC staining of CD38 of OSCC in TCs, FLCs, and TILs. **(C)** The IHC score of CD38 in TCs, FLCs, and TILs from OSCC patients. * and *** represented that differences were considered statistically significant with P < 0.05 and P < 0.001, respectively.

On TIMER database, compared with the adjacent normal tissues, CD38 mRNA was significantly upregulated in cervical and endocervical cancers (CESCs), colon adenocarcinoma (COAD), esophageal carcinoma (ESCA), head and neck cancer (HNSC) and kidney renal clear cell carcinoma (KIRC) and was significantly downregulated in kidney chromophobe (KICH) (**Figure 1A**). Thus, to validate CD38 expression in OSCC, the current study included 92 patients with OSCC. As shown in **Table 1**, TCs in 92 samples, TILs in 87 samples, and FLCs in 63 samples were identified. Typical low and high expression of the CD38 IHC staining was presented in **Figure 1B**. The IHC scores of CD38 in TCs, TILs, and FLCs were shown to be comparable in OSCC (**Figure 1C**).

**Table 1:** Association between CD38 expression and clinicopathological characteristics in OSCC patients.

	TCs					FLCs					TILs				
Gender															
Female	39	16 (41.0%)	23 (59.0%)	0.002	0.963	26	13 (50.0)	13 (50.0)	3.477	0.062	39	32 (82.1)	7 (17.9)	2.619	0.106
Male	53	22 (41.5%)	31 (58.5%)			37	10 (36.5)	27 (73.0)			48	32 (66.7)	16 (33.3)		
Age															
<60	27	11 (40.7%)	16 (59.3%)	0.005	0.944	21	9 (42.9)	12 (57.1)	0.548	0.58	27	21 (77.8)	6 (22.2)	0.358	0.55
≥60	65	27 (41.5%)	38 (58.5%)			42	14 (33.3)	28 (66.7)			60	43 (71.7)	17 (28.3)		
Smoking															
No	67	28 (41.8)	39 (58.2%)	0.024	0.877	41	17 (41.5)	24 (58.5)	1.244	0.265	64	44 (68.8)	20 (31.3)	2.884	0.089
Yes	25	10 (40.0)	15 (60.0)			22	6 (27.3)	16 (72.7)			23	20 (87.0)	3 (13.0)		
TNM Stage															
I–II	46	25 (54.3)	21 (45.7)	6.81	0.009*	28	11 (39.3)	17 (60.7)	0.322	0.57	44	32 (72.7)	12 (27.3)	0.013	0.91
III–IV	44	12 (27.3)	32 (72.7)			34	11 (32.4)	23 (67.6)			42	31 (73.3)	11 (26.2)		
Difference															
well	23	7 (30.4)	16 (69.6)	1.178	0.278	17	8 (47.1)	9 (52.9)	1.235	0.266	22	17 (77.3)	5 (22.7)	0.154	0.695
Moderate to poor	67	29 (43.3)	39 (56.7)			44	14 (31.8)	30 (68.2)			63	46 (73.0)	17 (27.0)		
WPOI															
1–3	51	24 (47.1)	27 (52.9)	1.777	0.335	32	18 (56.3)	14 (43.8)	9.856	0.002*	50	35 (70.0)	15 (30.0)	0.646	0.421
4–5	38	14 (36.8)	24 (63.2)			29	5 (17.2)	24 (82.8)			36	28 (77.8)	8 (22.2)		
Lymph node metastasis															
No	57	29 (50.9)	28 (49.1)	7.663	0.006*	37	14 (37.8)	23 (62.2)	0.005	0.637	53	40 (75.5)	13 (24.5)	0.457	0.499
Yes	33	7 (21.2)	26 (78.8)			25	9 (36.0)	16 (64.0)			32	22 (68.8)	10 (31.3)		

TCs, tumor cells; FLCs, fibroblast-like cells; TILs, tumor-infiltrating lymphocytes; WPOI, worst pattern of invasion; χ2, Pearson’s chi-squared test.

* represented that differences were considered statistically significant with P < 0.05.

### The Correlation Between CD38 Expression and Clinicopathological Characteristics as Well as Ki-67 Staining

**Figure 2:**
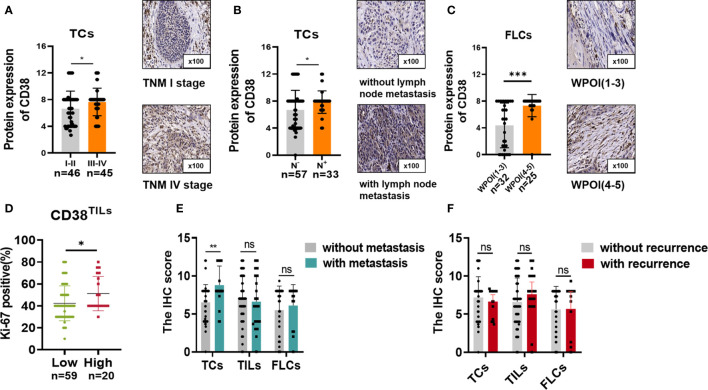
CD38 expression with **(A)** different TNM stages and **(B)** lymph node metastasis in TCs and **(C)** different WPOIs in FLCs. Correlation between Ki-67 staining and CD38^TILs^ levels **(D)**. Correlation between CD38 expression and metastasis status in TCs, TILs, and FLCs **(E)**. Correlation between CD38 expression and recurrence status in TCs, TILs, and TILs **(F)**. *,**,*** represented that differences were considered statistically significant with P < 0.05, P < 0.01 and P < 0.001 espectively, and ns reprented no statistical differences.

We further analyzed the correlation between the expression of CD38 and clinicopathological features of patients with OSCC (**Table 1**). The results demonstrated that the expression of CD38 had no significant relationship with gender, age, smoking habits, and differentiation. However, more presentation of CD38^TCs^ in OSCC patients was correlated with advanced TNM stage (**Figure 2A**) and higher risk of lymph node metastasis (**Figure 2B**).

In addition, upregulated CD38^FLCs^ was closely related to worse WPOI (**Figure 2C**). Since CD38 was found to promote cell proliferation in cervical cancer (18), it is suggested that cell proliferation is due to abnormally expressed CD38. Therefore, we conducted IHC staining of proliferation index Ki-67 and found that patients with increased CD38 in TILs had more Ki-67 staining of TCs (**Figure 2D**), suggesting that CD38 in TCs, TILs, and FLCs were all involved in OSCC progression in different ways.

### Upregulated CD38 Correlated With Higher Risk of Postoperative Metastasis and Poor Survival

**Figure 3:**
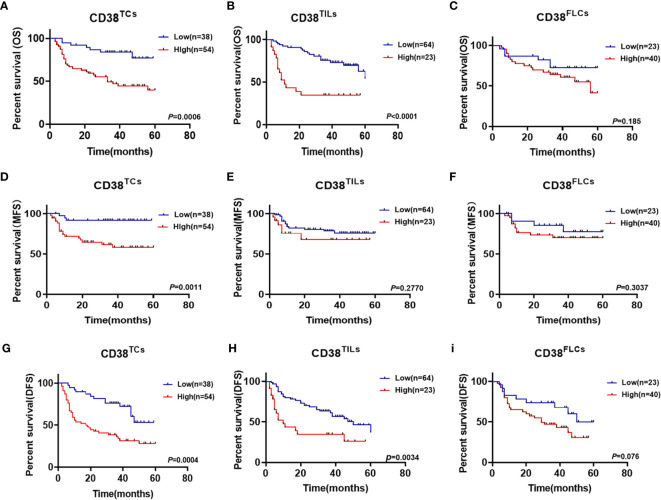
Kaplan–Meier survival curves for overall survival time **(A–C)**, metastasis free survival time **(D–F)** and disease-free survival time **(G**–**I)** of OSCC patients according to the expression of CD38 in TCs, TILs and FLCs.

Considering upregulated CD38 tends to have a malignant association with poor clinical outcomes. The association between CD38 and postoperative recurrence as well as postoperative metastasis status was further analyzed. Our results suggested that increased CD38^TCs^, instead of CD38^TILs^ and CD38^FLCs^, had a higher risk of postoperative metastasis after surgery (**Figure 2E**). Moreover, the relationships between recurrence and the CD38 expression were determined but showed no significance (**Figure 2F**).To confirm the prognostic value of CD38 to OSCC, Kaplan–Meier survival was used to analyze survival of OSCC patients included in this study. The data showed that enhanced CD38 in TCs had a shorter OS (**Figure 3A**), MFS (**Figure 3D**), and DFS (**Figure 3G**). Furthermore, OSCC patients with more CD38 in TILs had a shorter OS (**Figure 3B**) and DFS (**Figure 3H**), but MFS was found no significant difference in CD38 in TILs (**Figure 3E**). However, in FLCs, we found no significant difference in OS, RFS, and DFS (**Figures 3C, F, I**).

### Highly Expressed CD38TCs and CD38TILs Were Both Independent Prognostic Factors for OSCC

Univariate and multivariate Cox regression analyses were used to analyze the prognostic value of clinicopathological features. Our data confirmed that gender, age, smoking habits, TNM stage, WPOI, and CD38^FLCs^ had no significant predictive value for OS and DFS (all *P* > 0.05). But moderate-to-poor differentiation and high expression of CD38 in TCs and TILs were significantly independent prognostic indicators of OS and DFS for OSCC (**Table 2**).

**Table 2:** Cox-regression analysis of OS and DFS in OSCC patients.

Variables	Univariate analysis			Multivariate analysis		
OS	Hazard ratio	95% CI	P	Hazard ratio	95% CI	P
Gender						
Female *vs* Male	1.878	0.922–3.826	0.083			
Age						
≥60 *vs <*60	1.064	0.513–2.207	0.868			
Smoking						
Yes *vs* No	0.847	0.397–1.809	0.668			
TNM Stage						
III–IV *vs* I–II	2.011	1.021–3.963	0.043*	0.602	0.134–2.712	0.509
Differentiation						
Moderate to poor *vs* Well	5.445	1.633–18.154	0.006*	3.699	1.084–12.621	0.037*
WPOI						
4–5 *vs* 1–3	1.159	0.590–2.275	0.668			
Lymph node metastasis						
Yes *vs* No	2.193	1.137–4.236	0.019*	2.775	0.629–12.239	0.178
CD38 in TCs						
High *vs* Low	3.451	1.504–7.919	0.003*	2.639	1.076–6.474	0.034*
CD38 in FLCs						
High *vs* Low	1.856	0.729–4.725	0.195			
CD38 in TILs						
High *vs* Low	3.734	1.843–7.567	0.000*	3.328	1.597–6.935	0.001*
**DFS**						
Gender						
Female *vs* Male	1.517	0.850–2.705	0.158			
Age						
≥60 *vs*<60	1.105	0.596–2.050	0.751			
Smoking						
Yes *vs* No	1.234	0.662–2.304	0.508			
TNM Stage						
III–V *vs* I–II	1.926	1.088–3.409	0.025*	0.701	0.205–2.400	0.572
Differentiation						
Moderate to poor *vs* Well	2.813	1.251–6.324	0.012*	2.442	1.044–5.713	0.04*
WPOI						
4–5 vs 1–3	1.198	0.675–2.127	0.537			
Lymph node metastasis						
Yes *vs* o	2.224	1.271–3.891	0.005*	2.363	0.698–7.995	0.167
CD38 in TCs						
High *vs* Low	2.766	1.462–5.231	0.002*	2.408	1.173–4.942	0.017*
CD38 in FLCs						
High *vs* Low	1.97	0.908–4.271	0.086			
CD38 in TILs						
High *vs* Low	2.403	1.301–4.437	0.005*	2.024	1.053–3.892	0.034*

OS, overall survival time; DFS, disease-free survival time; CI, confidence interval; TCs, tumor cells; FLCs, fibroblast-like cells; TILs, tumor-infiltrating cells; WPOI, worst pattern of invasion.

* represented that differences were considered statistically significant with P < 0.05.

### Patients With High CD38 Levels in TILs Harbor Less CD3^+^ and CD4^+^ T Cell in Periphery Blood

**Figure 4:**
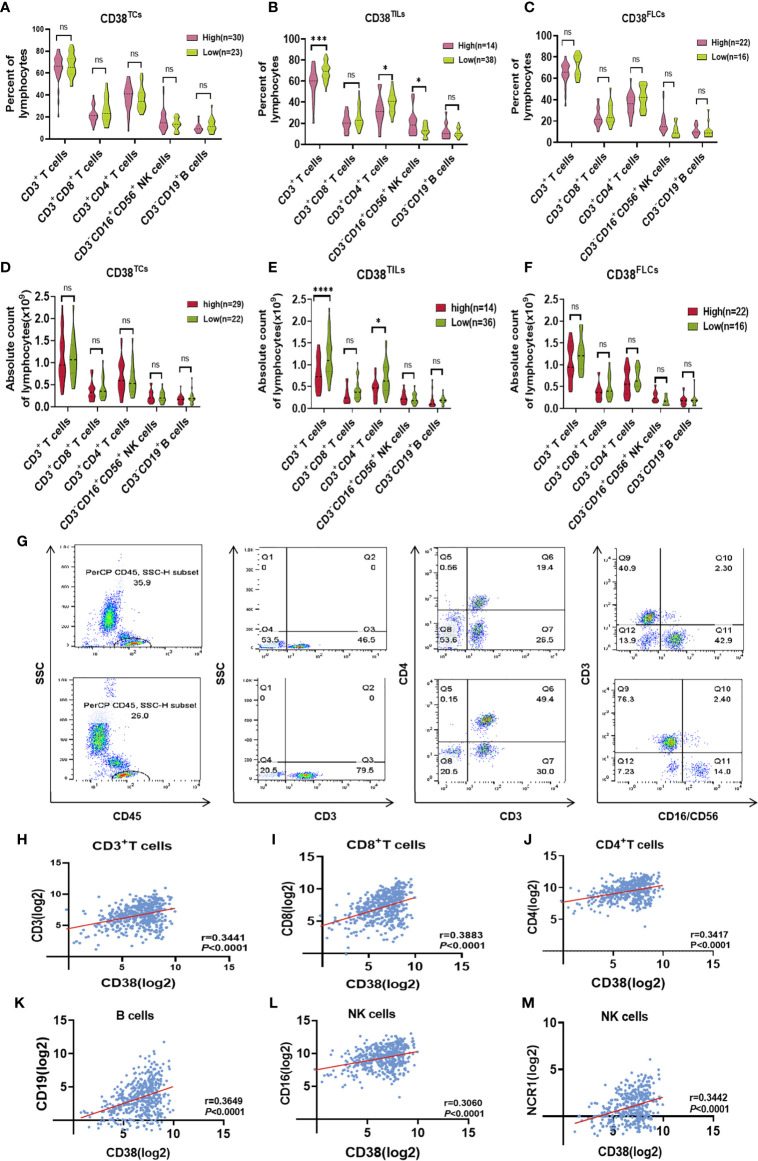
The change of lymphocytes subset in PBMC and tissue of OSCC patients according to CD38 level. The radio of lymphocytes subset of PBMCs in patients with distinct expression of CD38^TCs^ **(A)**, CD38^TILs^ **(B)**, CD38^FLCs^ **(C)**. The absolute count of lymphocytes subset of PBMCs in patients with distinct expression of CD38^TCs^ **(D)**, CD38^TILs^ **(E)**, CD38^FLCs^ **(F)**. **(G)**: Flow-cytometry dot plots show the strategy for gating lymphocytes, CD3^+^ T cells, CD3^+^CD4^+^ T cells and CD3^−^CD16^+^CD56^+^ NK cells with distinct expression of CD38 TILs. Correlation between CD38 and CD3^+^ T cells **(H)**, CD3^+^CD8^+^T cells **(I)**, CD3^+^CD4^+^T cells **(J)**, B cells **(K)**, NK cells **(L, M)** infiltration in HNSCC using cBioportal database. *,***,**** represented that differences were considered statistically significant with P < 0.05, P < 0.001 P < 0.0001 respectively, and ns reprented no statistical differences.

Our results revealed CD38 in TILs was a poor predictor for OSCC, but there was no correlation between CD38 and clinicopathological parameters. Considering CD38 also presents on NK cells, T cells and B cells in PBMCs, we proposed the hypothesis that CD38-positive immune subpopulations might dysregulate and negatively regulate in the immune system in the evolution and progression of OSCC. Thus, we used flow cytometry to analyze peripheral blood T, B, and NK cell proportion between CD38-low and CD38-high groups, and the strategy for gating lymphocytes was shown in **Figure 4G**. We found that patients with increased CD38^TILs^ had a lower proportion and absolute count of CD3^+^ T cell as well as CD3^+^CD4^+^T cells (**Figures 4B, E**) and a higher percentage of CD3^−^CD16^+^CD56^+^NK cells in PBMCs (**Figure 4B**), but the frequencies and number of lymphocyte subsets showed no difference between low and high subpopulation grouped by CD38^TCs^ (**Figures 4A, D**) and CD38FLCs (**Figures 4C, F**).

After confirming the correlation between CD38 and circulating lymphocyte subsets, we further performed a correlation analysis between CD38 and immune infiltration level in HNSCC microenvironment. The correlation between CD38 gene expression and TILs level was analyzed in the CBioPortal databse. Results showed that CD38 in HNSCC tissues was positively correlated with infiltrated CD3^+^ T cell (r=0.3441 p<0.001 **Figure 4H**), CD8^+^ T cell (r=0.3883 p<0.001 **Figure 4I**), CD4^+^ T cell (r=0.3417 p<0.001 **Figure 4J**), B cell (r=0.3649 p<0.001 **Figure 4K**) and NK cell (r=0.3060, r=3442 P<0.001 **Figures 4L, M**). In addition, TIMER was also used to further analyze. **Table 3** showed the correlation between CD38 and specific immune cell marker infiltration at transcription level. Partial correlation and correlation adjusted by tumor purity were also provided. We found CD38 was strongly related to important markers of various immune cells including CD8^+^ T cells, general T cells, B cells, monocytes, tumor-associated macrophages, M1 macrophages, M2 macrophages, neutrophils, natural killer cells, dendritic cells, Th1, Th2, follicular helper T cells, Th17. regulatory T cells and T cell exhaustion. The results revealed that CD38 was strongly linked to immune infiltration of HNSCC, indicating that CD38 in tumor microenvironment and circulating lymphocytes is linked to immune imbalance.

**Table 3:** Correlation analysis between CD38 and immune cell infiltrations in HNSCC samples using TIMER.

Description	Gene markers	CD38			
		None		Purity	
		Cor	P	Cor	P
CD8+ T cell	CD8A	0.416	***	0.416	***
	CD8B	0.358	***	0.383	***
T cell (general)	CD3D	0.361	***	0.361	***
	CD3E	0.421	***	0.428	***
	CD2	0.394	***	0.396	***
B cell	CD19	0.381	***	0.376	***
	CD79A	0.425	***	0.418	***
Monocyte	CD86	0.334	***	0.337	***
	CSF1R	0.325	***	0.332	***
TAM	CCL2	0.321	***	0.326	***
	CD68	0.296	***	0.313	***
	IL10	0.259	***	0.263	***
M1 Macrophage	NOS2	0.306	***	0.302	***
	IRF5	0.21	***	0.227	***
	PTGS2	0.123	**	0.116	***
M2 Macrophage	CD163	0.336	***	0.354	***
	VSIG4	0.29	***	0.306	***
	MS4A4A	0.331	***	0.345	***
Neutrophils	CEACAM8	0.081	0.0651	0.068	0.133
	ITGAM	0.358	***	0.365	***
	CCR7	0.366	***	0.364	***
Natural killer cell	KIR2DL1	0.287	***	0.288	***
	KIR2DL3	0.284	***	0.282	***
	KIR2DL4	0.363	***	0.37	***
	KIR3DL1	0.261	***	0.245	***
	KIR3DL2	0.359	***	0.366	***
	KIR3DL3	0.129	**	0.11	*
	KIR2DS4	0.217	***	0.206	***
Dendritic cell	HLA-DPB1	0.345	***	0.35	***
	HLA-DQB1	0.289	***	0.277	***
	HLA-DRA	0.387	***	0.392	***
	HLA-DPA1	0.39	***	0.394	***
	CD1C	0.177	***	0.17	***
	NRP1	0.193	***	0.204	***
	ITGAX	0.403	***	0.42	***
Th1	TBX21	0.425	***	0.423	***
	STAT4	0.359	***	0.355	***
	STAT1	0.321	***	0.32	***
	IFNG	0.339	***	0.327	***
	TNF	0.117	**	0.102	*
Th2	GATA3	0.213	***	0.193	***
	STAT6	0.261	***	0.262	***
	STAT5A	0.351	***	0.343	***
	IL13	0.23	***	0.225	***
Tfh	BCL6	0.309	***	0.32	***
Th17	STAT3	0.335	***	0.331	***
	IL17A	0.154	***	0.141	**
Treg	FOXP3	0.386	***	0.387	***
	CCR8	0.377	***	0.373	***
	STAT5B	0.334	***	0.336	***
	TGFB1	0.001	0.98	0.014	0.758
T cell exhaustion	PDCD1	0.409	***	0.408	***
	CTLA4	0.327	***	0.319	***
	LAG3	0.366	***	0.357	***
	HAVCR2	0.388	***	0.397	***
	GZMB	0.364	***	0.359	***

TIMER, Tumor Immune Estimation Resource; TAM, tumor-associated macrophage; Th, T helper cell; Tfh, follicular helper T cell; Treg, regulatory T cell; Cor, R value of Spearman’s correlation; None, correlation without adjustment. Purity, correlation adjusted by purity.

*,**,*** represented that differences were considered statistically significant with P < 0.05, P < 0.01 P < 0.001 respectively.

### Positive Correlation Between CD38 and Immune Checkpoint in HNSCC

**Figure 5:**
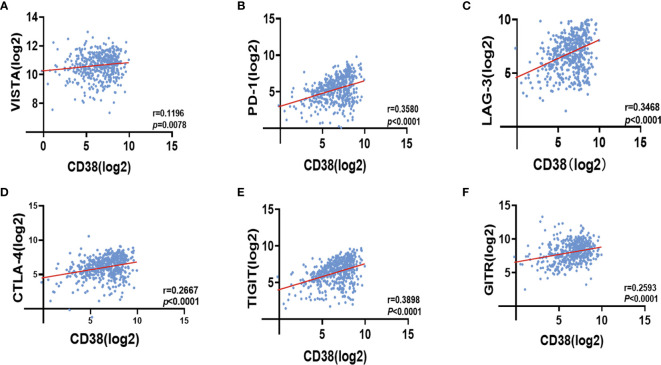
Correlation between CD38 expression and VISTA **(A)**, PD-1 **(B)**, LAG-3 **(C)**, CTLA-4 **(D)**, TIGIT **(E)** as well as GITR **(F)** in HNSCC with cBioportal database.

We found CD38^TILs^ was positively related to Ki-67 in TCs. At the transcriptional level, CD38 in tumor was positively correlated with infiltrated T cell exhaustion. Thus, we could speculate that CD38 in TILs may inhibit immune cell function to improve tumor proliferation. Because immune checkpoint proteins play a crucial role in the negative regulation of cellular immunity, we further analyzed the correlation between CD38 and immune checkpoint molecules by cBioportal. Interestingly, we found CD38 was positively linked to V-domain lg suppressor of T cell activation (VISTA r = 0.1196 **Figure 5A**), programmed death 1 (PD-1 r = 0.3580 **Figure 5B**), lymphocyte activation gene 3 (LAG-3 r = 0.3468 **Figure 5C**), cytotoxic T lymphocyte antigen 4 (CTLA-4 r = 0.2667 **Figure 5D**), T cell immunoglobulin and immunoreceptor tyrosine-based inhibitory motif (TIGIT r = 0.3898 **Figure 5E**), and glucocorticoid-induced tumor necrosis factor receptor (GITR r = 0.2593 **Figure 5F**).

## Discussion

Currently, the prognostic value of CD38 in OSCC had remained to be elucidated. The study demonstrated the expression pattern of CD38 for the first time, and high CD38 in TCs correlated with increased risk of metastasis. Moreover, high CD38^FLCs^ was found to have a worse WPOI, and higher expression of CD38^TILs^ was accompanied with higher Ki-67 staining. Interestingly, higher expression of CD38^TILs^ has more NK cells and less T cells, especially CD4^+^ T cells. In addition, CD38 in the tumor was positively correlated with infiltrated immune cell signatures in HNSCC. Strikingly, CD38 in HNSCC tumor tissues was positively related to immune checkpoint molecules. These results indicated that CD38 might promote tumor progression by regulating tumor growth, metastasis, and immune balance in OSCC.

CD38 research originally focused on hematological tumors, including CLL and multiple myeloma. However, with more research expanding the field, CD38 has been confirmed as expressed in multiple types of solid tumors such as nasopharyngeal cancer (19), cervical cancer (20), and skin cutaneous melanoma (21). CD38 is a non-lineage restricted glycoprotein expressed in both non-hematopoietic cells and diverse immune cells. In this study, the results showed that CD38 was widely expressed in TCs, FLCs, and TILs of OSSC samples. Notably, CD38 in these three cell types was expressed distinctively. Therefore, we concluded that CD38 in TCs, FLCs, and TILs was actually engaged in tumor progression, indicating that CD38 could not only regulate the behavior of tumor cells but also affect the tumor microenvironment.

In some solid tumors, such as nasopharyngeal carcinoma, upregulation of CD38 promoted tumor cell proliferation and metastasis (19). In cervical cancer cells, CD38 could promote cell proliferation and inhibit cell apoptosis (18). Previous studies found that CD38 was a novel regulator of CAFs’ pro-tumorigenic functions. Mechanistically, the knockout of CD38 decreased both CAF numbers and tumor growth. CD38 could increase fibroblastic migration towards tumor cells and enhance melanoma invasive behavior (22). In pancreatic ductal adenocarcinoma, CD38 was obvious increased on peripheral PD-1^+^CD8^+^T cells. High expression of CD38 in PD-1^+^CD8^+^ T lymphocytes subset was significantly associated with T, N, and M classification and TNM stage (23). A previous study demonstrated that the increased Ki-67 expression was found in CD38^+^ sub-populations from chronic lymphocytic leukemia cells (24). Consistently, in OSCC, we found that CD38^TCs^ significantly correlated with lymph node metastasis and postoperative metastasis. Besides, we found high expressed CD38^FLCs^ predicted a higher grade of WPOI. Additionally, elevated level of CD38^TILs^ was related to high Ki-67 of tumor cells. Insufficient tissues and individual differences in OSCC samples contributed to the absent CD38 staining of FLCs or TILs in some OSCC samples. However, this defect would not affect the expression of CD38 in TILs for two reasons. Firstly, the insufficient tissues and individual differences are inevitable, and excluding these samples in this study may cause selection bias. Secondly, only five samples did not have TIL score, accounting for 5.4% of the total sample, which have a very limited impact on the overall results.

A general consensus was reached that CD38 expression was a poor prognostic marker for chronic lymphocytic leukemia (CLL) (25). Similarly, hairy cell leukemia patients with higher CD38 expression were found to have shorter overall survival (26). However, skin cutaneous melanoma patients with increased CD38 expression showed a significantly higher survival rates of OS and DFS. Elevated CD38 in patients was associated with skin cutaneous melanoma metastasis (21). In our survival analysis, patients with high expression of CD38 in TCs had shorter OS, MFS, and DFS. Additionally, elevated CD38^TILs^ was correlated with shorter OS and DFS.

Cancer is not only a genetic disease but also an immunologic disease. The literature reported that numbers of cicrculating total CD3^+^, CD4^+^, and CD8^+^ T cell significantly raised with targeting CD38 treatment in multiple myeloma patients (27). In this study, we found that higher expression of CD38^TILs^ had lower percentage and absolute counts of CD3^+^ and CD3^+^CD4^+^ T cells, which suggested that CD38^TILs^ might affect CD3^+^ and CD3^+^CD4^+^ T cells to promote tumor growth and metastasis. Additionally, NK cells predominantly expressed CD38 in a constitutive manner (28). CD38 is also found to be physically/functionally aggregated with TCR, BCR, CD19 (T and B cell membrane molecules) (29). However, in this study, the ratio of CD3^−^CD16^+^CD56^+^ NK cells in blood was upregulated in patients with high CD38^TILs^, but CD19^+^ B cells showed comparable expression pattern within CD38-high and CD38-low patients. NK cells-derived CD38 in CD16^+^ NK cells is required for obtaining the effector cytotoxic phenotype (30). Importantly, immune cells in the tumor microenvironment were different from those in the peripheral blood. For example, the frequencies of CD38^+^HLA-DR^+^CD8^+^ T cells were enhanced in TILs compared to PBL in ovarian cancer and colorectal cancer patients (31). The results showed that the expression of CD38 in HNSCC was positively correlated with tumor infiltrating lymphocytes, indicating that CD38 exerted anti-tumor or pro-tumor effects depending on microenvironment property.

Recent years have witnessed the rapid development of tumor immunotherapy. Immune-checkpoint inhibitor (ICI) has become an important therapeutic backbone in recurrent/metastatic HNSCC (32). A recent study demonstrated that hepatocellular carcinoma patients with a high proportion of CD38^+^ immune cells achieved higher overall response rate of immune-checkpoint blockade therapy (33). We also discovered that CD38 expression positively correlated with PD-1, CTLA-4, TIGHIT, GITR, LAG-3, and VISTA, indicating that upregulated CD38 levels in tumor microenvironment mediated ICI-resistance. Indeed, CD38-mediated immunosuppression has been reported as a major mechanism of developed resistance to PD-1/PD-L1 blockade, and CD38 could inhibit CD8^+^ T cell function *via* adenosine receptor signaling (34).

In conclusion, we identified the tumorigenic-promoting role of CD38 in OSCC. Increased CD38^TCs^ had a higher risk for metastasis. Additionally, CD38^TCs^ and CD38^TILs^ can be used as potential biomarkers for OSCC. Furthermore, the present study provides new insight into CD38 immunosuppression, suggesting the inhibited immune function of CD38 was affected not only by tumor microenvironment but also through circulating lymphocytes. Future research is needed to unravel the role of CD38 in tumor promotion and immunomodulatory effect.

## Data Availability Statement

The raw data supporting the conclusions of this article will be made available by the authors, without undue reservation.

## Ethics Statement

The studies involving human participants were reviewed and approved by the Research Ethics Committee of Nanjing Stomatological Hospital. The patients/participants provided their written informed consent to participate in this study.

## Author Contributions

Data analysis, drafting, or revising the article were performed by all authors. LD and YN gave final approval of the version to be published, and QH was accountable for all aspects of the work. All authors contributed to the article and approved the submitted version.

## Funding

This work was supported by the National Natural Science Foundation of China (Grant No. 81902754, 82002865), Natural Science Foundation of Jiangsu Province (No. BK20190304, BE2020628), and Nanjing Medical Science and Technology Development Foundation, Nanjing Department of Health (No.YKK18123, YKK19091, YKK20151).

## Conflict of Interest

The authors declare that the research was conducted in the absence of any commercial or financial relationships that could be construed as a potential conflict of interest.
